# Lipid Metabolic Heterogeneity during Early Embryogenesis Revealed by Hyper-3D Stimulated Raman Imaging

**DOI:** 10.1021/cbmi.4c00055

**Published:** 2024-10-04

**Authors:** Jie Huang, Ling Zhang, Ninghui Shao, Yongqing Zhang, Yuyan Xu, Yihui Zhou, Delong Zhang, Jin Zhang, Hyeon Jeong Lee

**Affiliations:** † Zhejiang Polytechnic Institute, Polytechnic Institute, Zhejiang University, Hangzhou 310058, China; ‡ Liangzhu Laboratory, Zhejiang University, Hangzhou 311121, China; § Center for Stem Cell and Regenerative Medicine, Department of Basic Medical Sciences, and Bone Marrow Transplantation Center of the First Affiliated Hospital, Zhejiang University School of Medicine, Hangzhou 310058, China; ∥ College of Biomedical Engineering & Instrument Science, Key Laboratory for Biomedical Engineering of Ministry of Education, Zhejiang University, Hangzhou 310058, China; ⊥ Interdisciplinary Centre for Quantum Information, Zhejiang Province Key Laboratory of Quantum Technology and Device, Department of Physics, Zhejiang University, Hangzhou 310027, China; # MOE Frontier Science Center for Brain Science & Brain-Machine Integration, Zhejiang University, Hangzhou 310027, China; ∇ Center of Gene and Cell Therapy and Genome Medicine of Zhejiang Province, Hangzhou 310058, China

**Keywords:** stimulated Raman scattering, embryogenesis, lipid droplet, 3D imaging, lipid metabolism

## Abstract

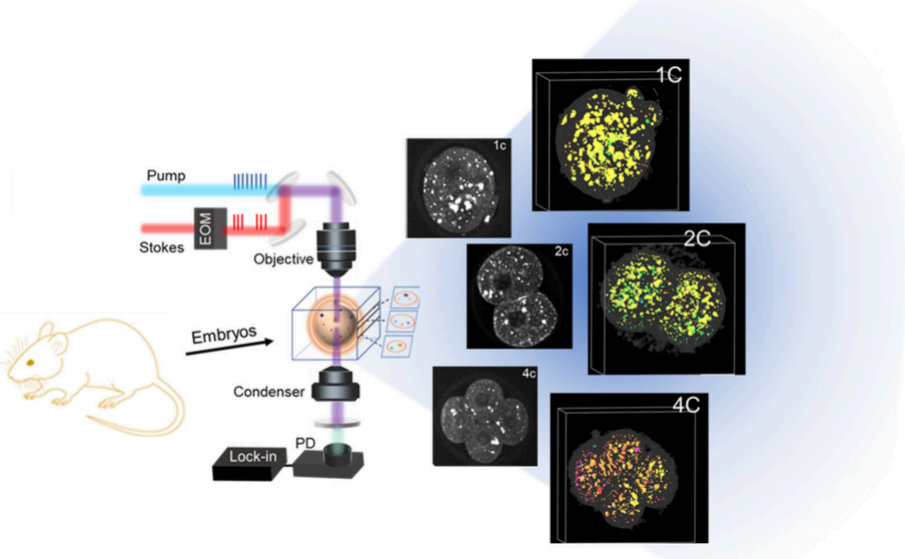

Studying embryogenesis is fundamental to understanding developmental biology and reproductive medicine. Its process requires precise spatiotemporal regulations in which lipid metabolism plays a crucial role. However, the spatial dynamics of lipid species at the subcellular level remains obscure due to technical limitations. To address this challenge, we developed a hyperspectral 3D imaging and analysis method based on stimulated Raman scattering microscopy (hyper-3D SRS) to quantitatively assess lipid profiles in individual embryos through submicrometer resolution (*x*–*y*), 3D optical sectioning (*z*), and chemical bond-selective (Ω) imaging. Using hyper-3D SRS, individual lipid droplets (LDs) in single cells were identified and quantified. Our findings revealed that the LD profiles within a single embryo are not uniform, even as early as the 2-cell stage. Notably, we also discovered a dynamic relationship between the LD size and unsaturation degree as embryos develop, indicating diverse lipid metabolism during early development. Furthermore, abnormal LDs were observed in oocytes of a progeria mouse model, suggesting that LDs could serve as a potential biomarker for assessing oocyte/embryo quality. Overall, our results highlight the potential of hyper-3D SRS as a noninvasive method for studying lipid content, composition, and subcellular distribution in embryos. This technique provides valuable insights into lipid metabolism during embryonic development and has the potential for clinical applications in evaluating oocyte/embryo quality.

## Introduction

Early embryogenesis is a dynamic phase characterized by rapid cellular transformations, with metabolism serving as a pivotal regulator.^1−3^ Among the metabolites, lipids are one of the central players due to their multifaceted roles in membrane integrity, organelle homeostasis,^4^ energy dynamics,^5^ and signaling pathways.^6^ The developmental potential of an embryo is intricately linked to the presence and quantity of specific fatty acid species,^7^ making the composition of fatty acids in follicular fluids or embryos a promising indicator of embryo quality^8^ and a prognostic marker for successful pregnancies following in vitro fertilization.^9^ Traditionally, lipid content in mammalian oocytes and embryos has been determined through destructive chemical analyses such as mass spectrometry.^10,11^ However, early stage embryos, composed of only a few cells, lack sufficient sample quantities for conventional analyses. Recent advancements in low-input and single-cell techniques have facilitated sequencing,^12−14^ generation of protein atlases,^15^ and metabolomic analysis,^3^ offering invaluable insights into the molecular regulatory mechanisms governing early embryogenesis. Nevertheless, these techniques are currently limited to a single cell or a small number of cells, leaving the dynamic information at the subcellular level elusive.

Lipid droplet (LD) is the reservoir for maintaining lipid homeostasis. Distinguished by their dynamic nature, LDs exhibit the capacity for altering the size, abundance, and intracellular localization. LDs are found ubiquitously within mammalian oocytes and preimplantation embryos, functioning as critical energy repositories.^16,17^ Their morphological dynamics are regulated by fluctuating cellular energy levels.^18^ Beyond their canonical role as lipid storage, LDs have been recognized as organelles bearing multifunctions. For instance, fatty acids hydrolyzed from LD, particularly polyunsaturated fatty acids, serve as precursors for eicosanoids and play regulatory roles in endocytosis/exocytosis and inhibition of ion channels and DNA polymerase.^19−21^ More recently, Arena et el. unveiled the indispensability of LDs in sustaining embryonic diapause,^22^ a pivotal reproductive strategy fostering species survival. Moreover, the intricate LD dynamics, including fusion and lipid mobilization, emerge as pivotal determinants of morphogenesis during the peri-implantation phase of embryonic development,^23^ highlighting the significance of imaging LD morphological features at the single-cell level.

Compared to exogenous labeling of LDs, which offers limited compositional or physiochemical information, vibrational spectroscopic imaging techniques, particularly Raman-spectroscopy-based methods, are increasingly recognized as unique tools for investigating biomolecules within their native microenvironments. Confocal Raman microscopy has successfully imaged LDs in mouse oocytes^24,25^ and embryos.^26^ Nevertheless, spontaneous Raman scattering is a weak process and requires a long data acquisition time (typically ∼3.5 h for a 2D image). Third harmonic generation (THG) microscopy has been employed for label-free LD imaging in mouse embryos,^27^ but THG is sensitive to interfaces rather than chemical composition, making it challenging for accurate analysis of lipid quantity and types. Recently, coherent anti-Stokes Raman scattering (CARS) imaging has been utilized to quantify the size, number, and spatial distribution of LDs in mouse embryos^28^ and model organisms.^29^ However, CARS imaging has a quadratic concentration dependence and spectral distortions due to a significant nonresonant background, hindering quantitative analysis of lipid compositions. On the other hand, stimulated Raman scattering (SRS)^30^ intensity is linearly proportional to molecular concentration. SRS at C–H stretching frequency has been used to image a single plane of the cells or spheroids to quantify lipid content and unsaturation.^31−33^ More recently, advances in hyperspectral SRS imaging and analysis methods have enabled the simultaneous mapping of multiple biomolecules.^34,35^ Furthermore, deuterium incorporation into LDs and other biomolecules has facilitated tracking metabolic activities and dynamics in various disease models through SRS imaging.^36−38^

**Figure 1:**
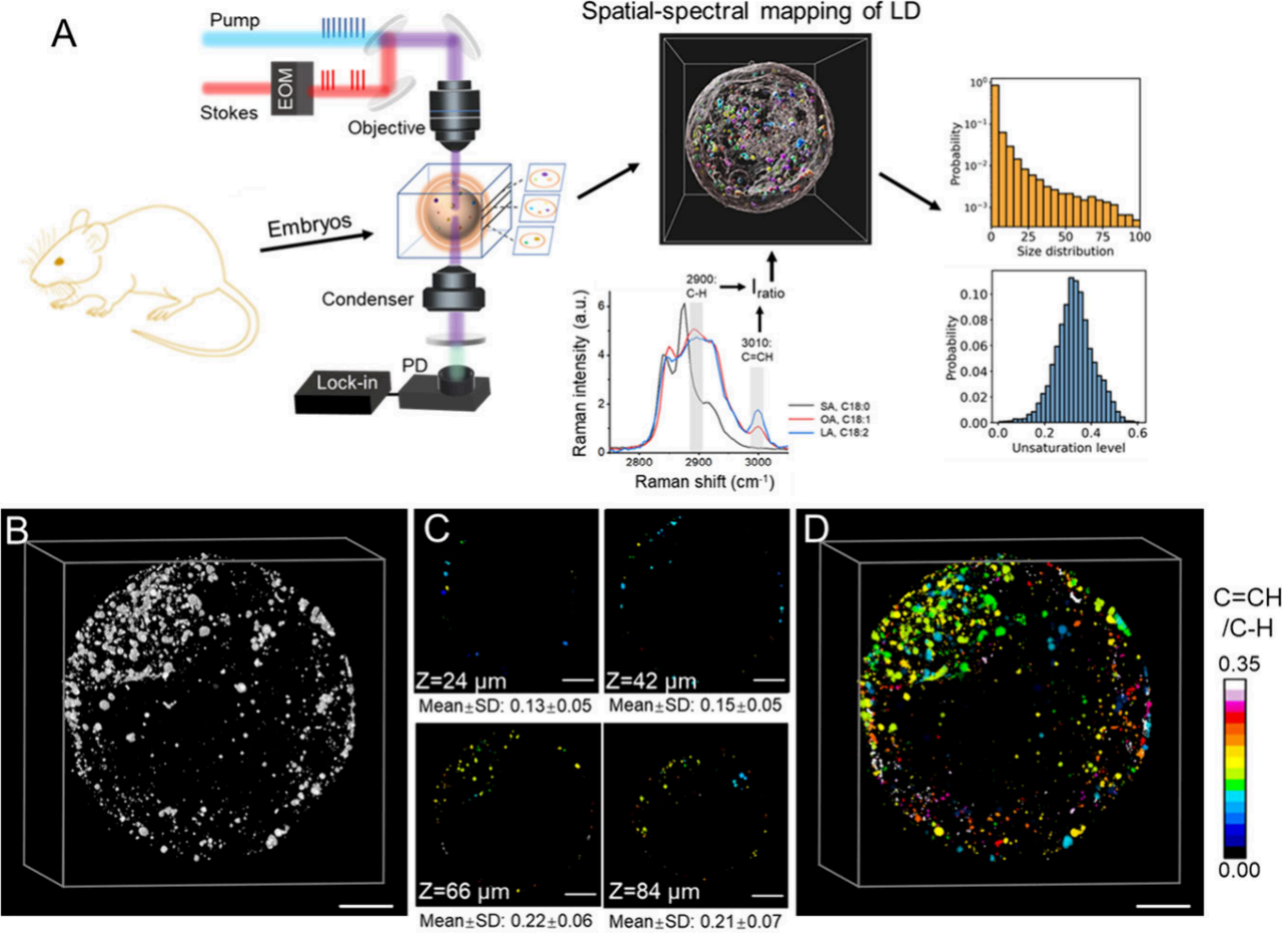
Hyper-3D SRS imaging of mouse embryos for quantitative LD analysis. (A) Schematics of 4D SRS imaging and analysis. (B) Raw 3D SRS image at 2900 cm^–1^. (C) Lipid unsaturation level at different depths. (D) 3D distribution of LDs with various sizes and unsaturation levels in the whole embryo. Scale bar: 20 μm.

In this work, we present a volumetric hyperspectral stimulated Raman scattering (SRS) imaging and analysis method (hyper-3D SRS) for 3D chemical imaging of LDs in single cells during early embryogenesis (Figure 1A). With high spatial and spectral resolutions for lipid analysis, hyper-3D SRS imaging of early stage embryos revealed previously unknown correlations between LD size and the unsaturation level at specific stages of embryo development. Moreover, we observed that LD aggregation size significantly differs between the two cells in the 2-cell stage embryo, suggesting the establishment of asymmetry in the lipid metabolic pathway as early as the 2-cell stage. These findings exemplify the capabilities of hyper-3D SRS in studying LD dynamics in spatial and spectral domains, providing novel insights into mechanisms that cannot be obtained by using conventional methods. To demonstrate the clinical applications, we compared the lipid signatures of oocytes from a progeria model and identified abnormal LDs in progeria oocytes, suggesting the LD property as a potential biomarker for evaluating oocyte/embryo quality. Overall, this study demonstrates the unique capabilities of hyper-3D SRS in tracking lipid dynamics at the subcellular level, providing valuable insights into lipid metabolism in a complete 3D biological system.

## Results

### Single LD Compositional Analysis by Hyper-3D SRS Reveals Heterogeneity in Lipid Unsaturation Levels across the Whole Embryo

To visualize lipids in embryos, we tuned the laser beating frequency to be resonant with 2800–3050 cm^–1^, corresponding to C–H vibrations. By focusing on 2900 cm^–1^, primarily associated with C–H stretching vibrations from lipids,^39^ the morphological outline of the whole embryo at the blastocyst (BC) stage was visualized (Figure S1). Compared to bright field imaging, SRS provided lipid-selective imaging from which a large number of LDs were observed (Figure S1A–C). By adjusting the sample stage for *z*-axis scanning, the 3D distribution of LDs was imaged, showing an aggregation of LDs in the inner cell mass, with a few LDs present in the trophoblast (Figure 1B). Notably, the composition of individual LDs was resolved by hyperspectral SRS imaging, showing the presence of unsaturated lipids, as indicated by the peak at 3010 cm^–1^, representing C=CH bonds^40^ (Figure S1D,E). As the SRS intensity at 3010 cm^–1^ directly correlates with the number of C=C bonds (Figure S2A), the unsaturation level of single LDs can be quantified by normalizing the 3010 cm^–1^ peak with another peak representing total C–H bonds, such as 2900 and 1445 cm^–1^ representing C–H stretch vibrations in all of the lipids^39^ and the CH_2_ bending mode,^41^ respectively. Both the 3010/2900 cm^–1^ and the 3010/1445 cm^–1^ ratios showed a linear correlation with the number of C=C bonds in the fatty acid standards (Figure S2B and S2C), validating the quantitative approach for mapping lipid unsaturation levels. Due to the stronger SRS signal at 2900 cm^–1^ compared to 1445 cm^–1^, we adopted a 3010/2900 cm^–1^ ratio for imaging and quantifying the unsaturation level of LDs at different *z*-layers of an embryo (Figure 1C). While the 2850 cm^–1^ peak is commonly used to represent lipids, we observed that the 2900 cm^–1^ intensity remained more stable (Figure S2B). This may be due to the 2850 cm^–1^ peak being influenced by lipid properties, such as the number of CH_2_ chains, which could complicate quantification. The selection of the 2900 cm^–1^ peak for normalizing lipid signals in unsaturation quantification has also been demonstrated previously.^42^ The hyperspectral images revealed significant variation in the unsaturation level of LDs across different layers (Figure 1C), emphasizing the importance of obtaining complete 3D compositional information to assess the LD dynamics during embryogenesis.

To extract the multidimensional information from a single embryo for LD dynamics, we developed a hyper-3D SRS imaging and analysis method (Figure S3). Focusing on the degree of unsaturation of LD, SRS images of oocytes and embryos were acquired at 3010 and 2900 cm^–1^ for quantifying the unsaturation level of each pixel by calculating the ratio of these two images. LDs were segmented based on their strong SRS signal at 2900 cm^–1^, which arises from a significantly higher CH chain concentration than the cytoplasm. Instead of applying global thresholding, we utilized a Bradley adaptive threshold^43^ to account for spatial variations in the signal resulting from laser scanning. While SRS signals are linearly correlated with molecular concentration,^30^ it is known that the signal intensity attenuates considerably with increasing imaging depth,^44^ as observed in the single-frequency SRS images of the oil film (Figure S4A–D). However, this artifact is eliminated when the ratio of the two SRS images is calculated, providing a consistent measure of unsaturation over ∼70 μm (Figure S4E,F). This result indicates the feasibility of quantitatively analyzing the spatial and spectral information on LDs in the 3D images, within the range of 70 μm thickness, which is appropriate for mouse oocytes or embryos (70–100 μm). Following the reconstruction of the 3D distribution of LDs with various unsaturation levels, LD quantifications were performed on volume, number, and unsaturation level, revealing a high degree of heterogeneity in lipid size and unsaturation level throughout the entire embryo (Figure 1D).

### Quantitative Evaluation of LD Unsaturation by Hyper-3D SRS Imaging of Mouse Embryos

**Figure 2:**
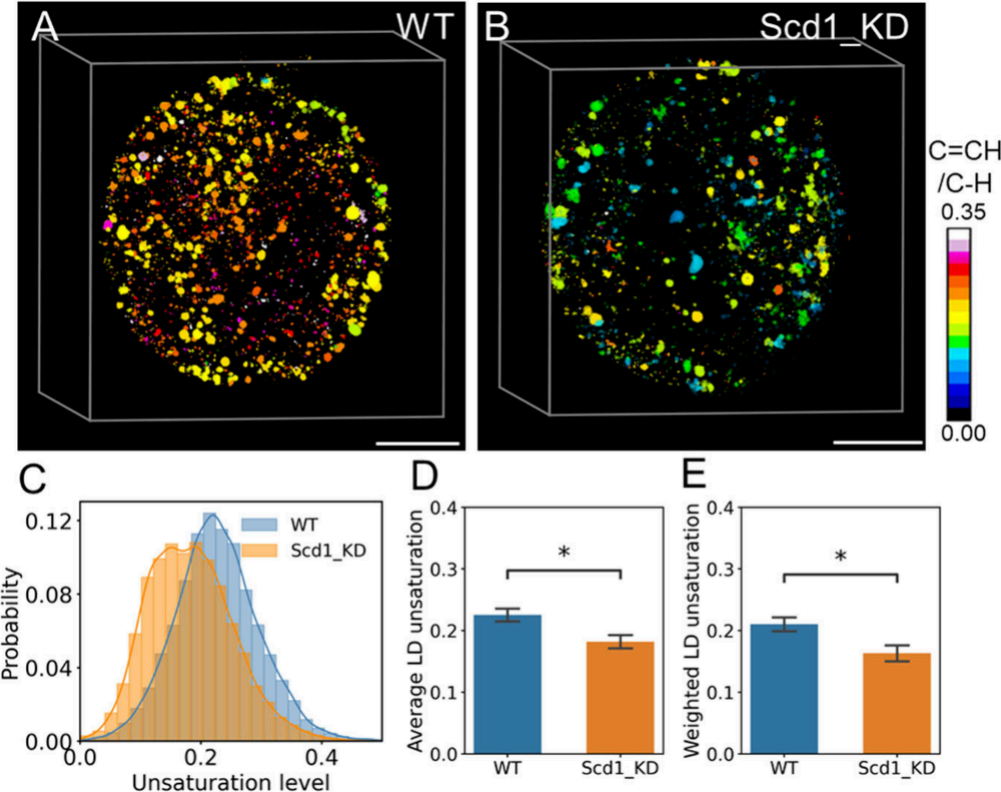
Reduction of lipid unsaturation level in embryo after Scd1 knockdown quantified by hyper-3D SRS. (A) 3D spatial distribution of LDs unsaturation level in BC. (B) 3D spatial distribution of LDs unsaturation level in BC with Scd1 knockdown. (C) Frequency distribution for unsaturation levels of individual LDs from all embryos. (D) Average unsaturation of LDs between the two groups. (E) Unsaturation of LDs between two groups of embryos. WT, *n* = 11; Scd1_KD, *n* = 10. Scale bar, 20 μm. *P < 0.05, data shown as mean ± standard error of the mean (SEM).

To validate the quantification of the unsaturation level by hyper-3D SRS, we knocked down stearoyl-CoA desaturate 1 (Scd1_KD), the key enzyme catalyzing the synthesis of monounsaturated fatty acids, and compared the unsaturation levels quantified by hyper-3D SRS imaging of BC stage embryos (Figure 2). When Scd1 is suppressed, the unsaturation level of LDs is reduced significantly in the entire embryo (Figure 2A,B). Statistical analysis further verified a consistent reduction of unsaturation level in multiple embryos (Figure 2C), indicating about 25% reduction of the average unsaturation level of LDs in the Scd1_KD group compared to wild type (Figure 2D). To eliminate the possibility of influence coming from the LD volume in the process of calculating average unsaturation of LDs, we quantified the weighted unsaturation level of LD with the volume of LD as the weight (Figure 2E), which also showed a similar reduction of unsaturation. The quantification of other LD features, such as number and volume, showed no significant change in the Scd1_KD group compared to the wild type (Figure S5). These results demonstrate that hyper-3D SRS imaging can quantitatively extract compositional information, such as unsaturation at the subcellular level, in a single embryo.

### Single LD Analysis at the Single-Cell Level Shows Establishment of Asymmetry in LD as Early as in the 2C Stage

**Figure 3:**
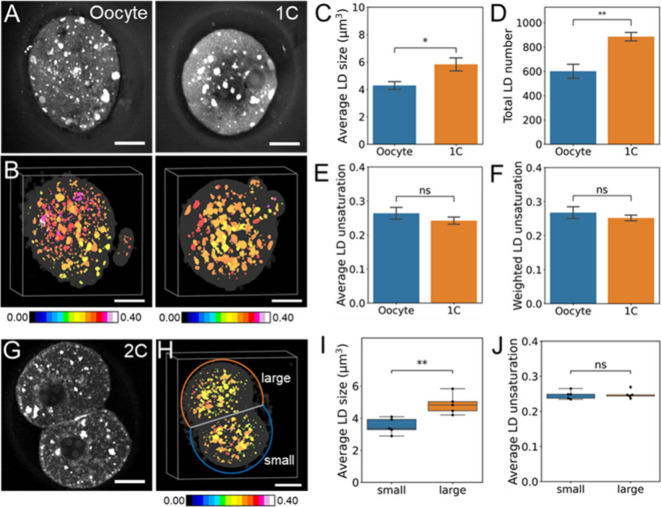
Distinct LD profiles upon fertilization and between 2 cells at 2C stage embryo revealed by hyper-3D SRS imaging of LD in single cells. (A) The raw SRS image at 2900 cm^–1^ at oocyte and 1C stage. (B) Representative 3D lipid unsaturation level distribution. (C) Average LD size per cell, (D) LD number per cell, (E) average LD unsaturation per cell, and (F) weighted LD unsaturation per cell of the oocyte and 1C stage. Oocyte: *n* = 6; 1C, *n* = 5. (G) The raw SRS image at 2850 cm^–1^ (lipid) at the 2C stage. (H) Representative 3D lipid unsaturation level distribution at the 2C stage. (I,J) Comparison of LD distribution of two cells at the 2C stage. Each point represents quantification from a single cell, *n* = 5. n.s., nonsignificant, *P < 0.05, **P < 0.01, data shown as mean ± sem. Scale bar: 20 μm.

It was long-believed that the individual cell in the embryo of the early developmental stage is identical.^45,46^ However, more recent studies from single-cell sequencing have demonstrated that cells start showing heterogeneity as early as in the 4-cell stage (4C) or even the 2-cell stage (2C),^47−49^ indicating the breaking of embryo symmetry happens much earlier than establishment of cell polarity at the 8-cell stage (8C).^50−52^ However, whether asymmetry in the metabolism happens in the embryo remains unknown, which requires an in situ metabolic measurement of individual cells in the embryo. To demonstrate the capability of measuring LD profiles at the single-cell level, we performed hyper-3D SRS imaging of oocyte and zygote (1C) and developed a 3D cell segmentation method to automatically segment single cells from the SRS image at C–H stretch vibrations (Figure S6). The LD volume, number, and degree of unsaturation were quantified from SRS signals at 3010 and 2900 cm^–1^ (Figure 3A,B). Compared to the oocyte, both LD size and number increased in 1C (Figure 3C,D), indicating increased lipid reservoir after fertilization. At the same time, a slight reduction of unsaturation level was found in 1C compared to oocyte (Figure 3E,F), reflecting the alteration of lipid compositions after fertilization.

To test whether metabolic asymmetry happens at 2C, we analyzed LD profiles of individual cells in 2C embryo using the single-cell segmented hyper-3D SRS approach (Figure 3G,H). Comparing the two cells, it was found that one cell consistently shows larger LD aggregates compared to the other, while the LD numbers do not differ significantly (Figures 3I and S6). The unsaturation level of LDs did not show an obvious difference between the two cells (Figure 3J). Interestingly, although not statistically different, the two cells can be divided into large and small cells based on the slight variations of their volumes (Figure S6), where larger LD aggregates were found in the large cell. These results suggest that mouse embryos start showing asymmetry in the balance between lipid synthesis and utilization as early as 2C, providing new insight into embryonic metabolism.

### Hyper-3D SRS Imaging Shows LD Dynamics during Early Embryonic Development

**Figure 4:**
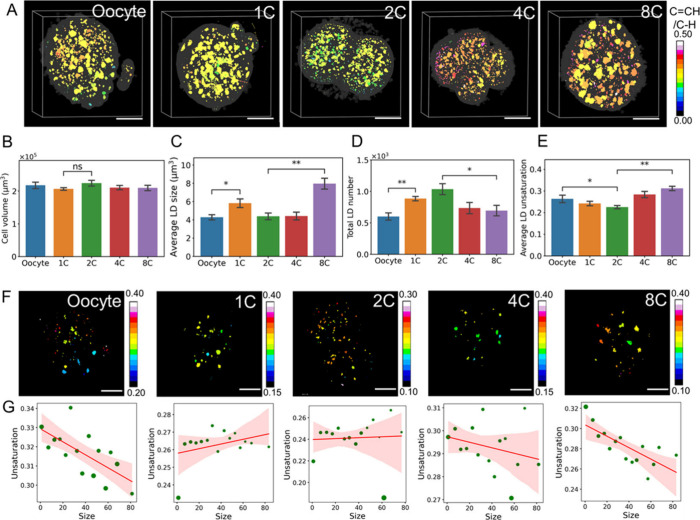
Dynamic changes in size and unsaturation level of LDs in early embryonic development. (A) Representative 3D lipid unsaturation level distribution from different developmental stages. (B) Cell volume, (C) LD size, (D) number of LDs, and (E) average unsaturation of LDs in oocytes and early embryonic stages. (F) Representative heat maps of the lipid unsaturation level. (G) Correlation between size of LD aggregates and unsaturation level at different embryonic stages. The line and shading in the graph represent the linear regression and 95% confidence interval, respectively. Oocyte, *n* = 6; 1C, *n* = 11; 2C, *n* = 5; 4C, *n* = 8; 8C, *n* = 7. n.s., nonsignificant, *P < 0.05, **P < 0.01. Scale bar: 20 μm.

To investigate LD dynamics during early embryogenesis, we tracked physiochemical properties of LDs by hyper-3D SRS from oocyte, 1C, 2C, 4-cell (4C), to 8-cell (8C) embryos (Figure 4A). The embryo volume remained constant (Figure 4B). Although a substantial number of LDs was found in all stages of embryos, LD number and size showed distinct features for different stages (Figure S7A). Specifically, in addition to a sudden increase in LD volume from oocyte to 1C as described in Figure 3, 8C also showed significantly larger LD aggregates (Figure 4C), which may be related to the generation of morphological compaction and polarity in this period. The LD number increased gradually until 2C, followed by a reduced LD number in 4C and 8C (Figure 4D).

Hyper-3D SRS imaging further showed alteration in the unsaturation level of LDs at different stages (Figure S7B). The average unsaturation level of LDs was reduced from oocyte to 2C, followed by an increase from 4C to 8C (Figure 4E). Hyperspectral SRS imaging and quantification of individual LD further verified the quantitative evaluation of the unsaturation level of LDs by hyper-3D SRS (Figure S8). Analyzing the multidimensional information obtained from hyper-3D SRS, we noticed that only certain stages of the embryo exhibit a correlation between the size of the LD aggregates and the unsaturation level. Specifically, in oocyte and 8C, a smaller LD contains a higher unsaturation level; while other stages of embryos (1C, 2C, and 4C) show no or weak correlation (Figure 4F,G). It is interesting to note that fertilization has significantly reduced the unsaturation level of LDs, and as the embryo develops into 8C, the unsaturation level of LDs increases again with smaller LDs containing a high degree of unsaturation, suggesting increased unsaturated lipid synthesis. These data demonstrate the capability of hyper-3D SRS to study LD dynamics in spatial and spectral domains, providing insights on mechanisms that are difficult to obtain by conventional methods.

### LD Size and Unsaturation Level in Oocyte Were Identified as Potential Signatures for Aging and Reduced Blastocyst Formation Rate

**Figure 5:**
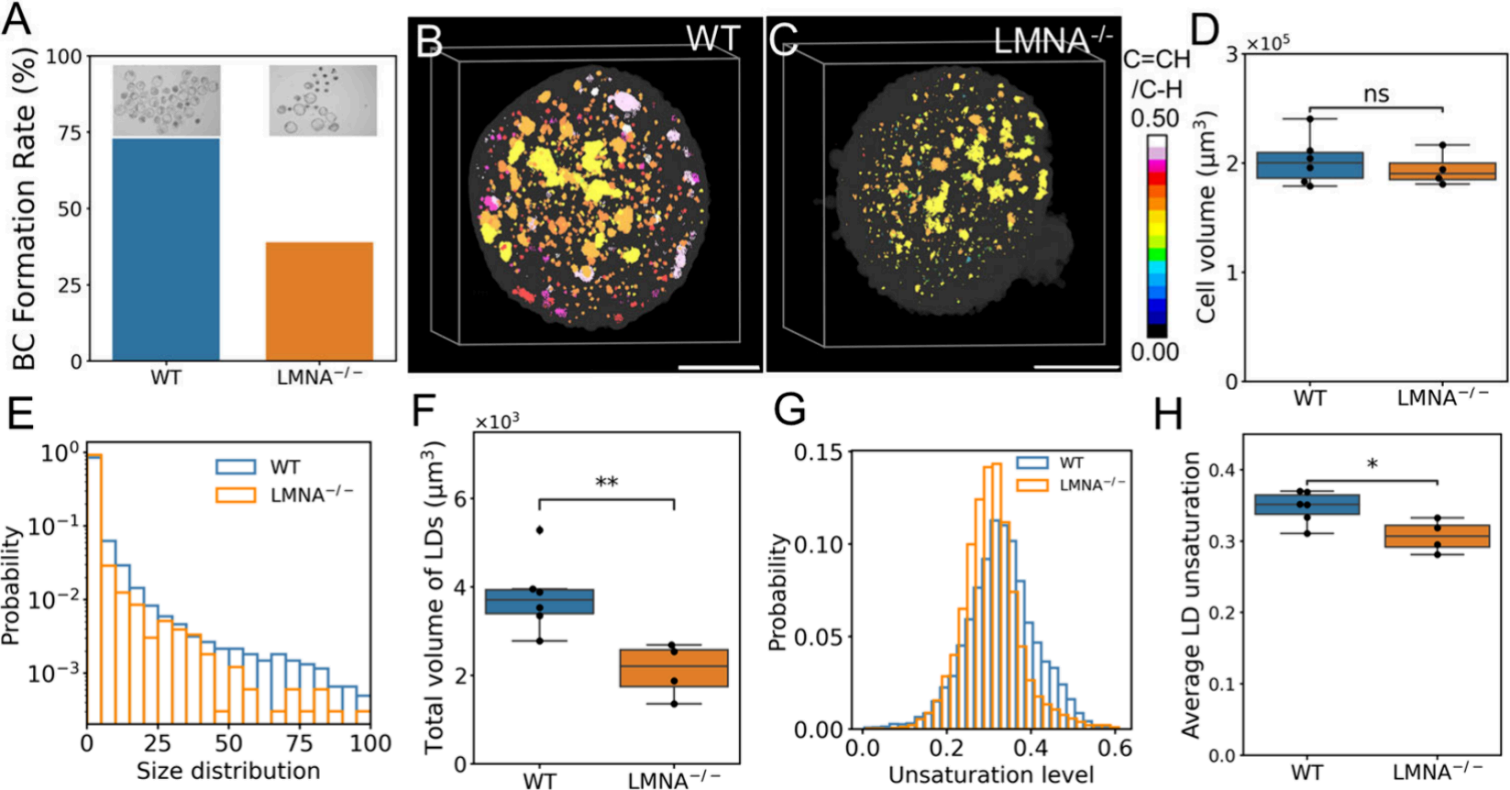
Changes in lipid metabolism of oocytes in progeria model (LMNA^–/–^). (A) The blastocyst formation rate of normal and progeria oocytes after fertilization. (B) 3D lipid unsaturation level distribution in a representative normal oocyte. (C) 3D lipid unsaturation level distribution in representative progeria oocyte. (D) Cell volume in normal and progeria oocytes. WT, *n* = 6; LMNA^–/–^, *n* = 4. (E) LD size distribution of normal and progeria oocytes. (F) Total volume of LDs in normal and progeria oocytes. (G) Frequency distribution of LDs unsaturation level of normal and progeria oocytes. (H) Average unsaturation of LDs in normal and progeria oocytes. Each point represents one cell, Mann–Whitney U-test, n.s., nonsignificant, *P < 0.05, **P < 0.01, data shown as mean ± sem. Scale bar: 20 μm.

With the noninvasive nature of hyper-3D SRS imaging in measuring lipid profiles, we sought to demonstrate the potential of using this technique for identifying molecular biomarkers of unhealthy oocytes. The aging process is known to adversely affect the quality of oocytes, impacting both pre- and postimplantation development.^53^ Using a progeria mouse model generated by Lamin A mutation, we found that the blastocyst formation rate is reduced in oocytes with aging conditions (Figure 5A). To study the spatiospectral changes of lipids, hyper-3D SRS imaging was performed on the oocytes (Figure 5B,C). Notably, the volume of oocytes showed no significant difference between wild-type and progeria models (Figure 5D), indicating that morphological assessment alone may be insufficient to accurately distinguish oocytes affected by aging.

By performing a single LD analysis, we found that, although the number of LD aggregates remained similar between progeria and wild-type oocytes (Figure S9A), their size (Figures 5E and S9B) and total lipid volume (Figure 5F) were significantly reduced in the progeria model. Moreover, the unsaturation level of individual LDs was markedly lower in progeria compared to wild-type oocytes (Figure 5G,H). We note that when evaluating the overall unsaturation level of oocytes by calculating the weighted unsaturation from LDs within each oocyte, the difference between wild-type and progeria model becomes smaller (Figure S9C). This is attributed to the lower average unsaturation level in wild-type oocytes when accounting for LD volume, likely due to the presence of smaller LDs with higher unsaturation levels, as shown in Figure 4E. Collectively, these results suggest that spatial-spectral dynamics of lipids, including LD size, amount, and unsaturation level, could serve as biomarkers for oocyte conditions, which can be noninvasively probed by hyper-3D SRS for oocyte/embryo quality evaluation.

## Discussion

The precise equilibrium between lipogenesis and lipolysis/lipoautophagy plays a pivotal role in embryonic development. LD is an organelle that reflects this delicate balance, with its number, size, and composition serving as valuable indicators. An imbalance in LD content has been shown to reduce oocyte/embryo developmental competence, especially during early embryonic stages.^54^ However, quantitatively analyzing LD properties, including the in situ compositional measurement of individual LDs, has presented significant challenges. In this study, we demonstrated multispectral SRS imaging as a unique tool for 3D visualization of lipid content and composition within oocytes and embryos, achieving submicrometer spatial resolution. Importantly, this approach offers noninvasive and chemically specific tracking. As a result, individual LDs were quantitatively assessed, including their total number, size, and degree of unsaturation, all visualized in a 3D context.

Morphologically, the LD is highly diverse in size, which is tightly regulated by either local partitioning/synthesis or fusion with existing LDs. In the early stage of the embryo, we observed the aggregation of small LDs. This may suggest active lipogenesis and lipolysis activities, as most cells tend to increase the number of LDs when there is an increase in lipids^55,56^ and smaller LDs can be more efficiently hydrolyzed with more surface area for lipases.^57^ Intriguingly, single-cell analysis within an embryo revealed that the size of the lipid aggregates was relatively larger in the larger cell of the 2C stage embryo. This suggests that at the 2C stage differences in lipid metabolism between the cells have already commenced. This divergence in lipid dynamics could potentially lead to influence the fate of these cells during the subsequent stages. Based on this intriguing observation, further investigation into symmetry breaking during embryogenesis is crucial. Combining single-cell techniques,^58^ such as single-cell sequencing, CRISPR screening, and optogenetic approaches, could elucidate whether these metabolic differences stem from alterations in lipogenesis, lipolysis, or a combination of both. These studies could shed light on the intricate regulation of cell fate determination during early embryogenesis.

The compositional heterogeneity within individual LDs during embryogenesis remains relatively unexplored due to technical barriers. Hyper-3D SRS has uncovered a dynamic correlation between the size of the LD aggregates and unsaturation levels at various embryogenic stages. Specifically, a trend of increasing lipid unsaturation levels following the 2C stage, and a stronger correlation between the size of lipid aggregates and unsaturation levels toward the 8C stage, suggest an upregulation in the synthesis of unsaturated fatty acids, replenishing the pool of unsaturated lipids as the embryo develops. Recent studies have reported that phospholipid unsaturation increases during embryo development, particularly into the blastocyst stage, mediated by SCD1.^59^ Based on our results, we hypothesize that the increased synthesis of unsaturated fatty acids plays a crucial role in regulating membrane fluidity, which is essential for early embryogenesis by promoting the formation of apical domains.^60,61^ Future investigations, including studies combining genetic modifications, would provide deeper insights into lipid metabolism during early embryogenesis. While hyper-3D SRS offers valuable information, other imaging techniques, such as autofluorescence and fluorescence lifetime imaging, can effectively probe metabolic heterogeneity by measuring NADH/FAD molecules. These techniques are particularly useful for identifying metabolically distinct subpopulations, such as those responding to chemotherapy, making them powerful tools for assessing metabolic diversity in cells.^62^ Ultimately, the integration of multiple chemical imaging modalities could further elucidate lipid metabolism in early embryogenesis by capturing complementary aspects of metabolic processes.

Finally, the potential clinical application of the hyper-3D SRS technique was explored in this study. Noninvasive assessment of embryo quality is of significant relevance in IVF. Using a progeria model, we have identified potential metabolic markers in mouse oocytes that correlate with reduced blastocyst formation rates. Indeed, high unsaturation levels, provided by polyunsaturated fatty acids, are essential for oocyte meiosis and maturation.^63^ It is important to note that while mouse models provide valuable insights, there are inherent differences in the metabolic profiles and developmental dynamics between mouse and human oocytes/embryos. These differences emphasize the need for further validation in human samples prior to clinical translation. Nevertheless, hyper-3D SRS imaging provides subcellular-level molecular insights without any labeling or extraction, making it a promising tool for the clinical evaluation of human oocytes or embryos in a clinical setting. Moreover, the versatile capabilities of hyper-3D SRS can be extended to the monitoring of clinical samples, potentially advancing precision medicine in reproductive health.

## Methods

### Mouse Gamete and Embryo Collection

C57BL/6J mice were housed in the animal facility of Zhejiang University. They were maintained on a 12 h light and 12 h dark cycle. The catalog number of the diet is 1010085 (Xietong). All experimental procedures were performed in accordance with the Animal Research Committee guidelines of Zhejiang University. To obtain oocytes or embryos, 4-week-old C57BL/6J female mice were intraperitoneally injected with 7.5 IU of pregnant mare serum gonadotropin (San-Sheng Pharmaceutical) 48 h after injection of 7.5 IU of hCG (San-Sheng Pharmaceutical). The superovulated female mice were mated with C57BL/6J adult male mice overnight after hCG administration. Oocytes and embryos were flushed from the reproductive tract at defined time periods after hCG administration: 14 h (MII), 24 h (zygote), 48 h (2-cell), 54 h (4-cell), 68 h (8-cell), and 92 h (blastocysts).

### In Vitro Culture of Mouse Embryos

LMNA^–/–^ mice were obtained from the Jackson Lab. For culturing mouse embryos, PN5-stage zygotes from normal mice and LMNA^–/–^ mice at the same age were collected at 24 h after hCG administration and cultured in drops of indicated medium (KSOM (Millipore)) covered by a layer of mineral oil in a humidified incubator at 37 °C, under 5% CO_2_. The blastocyst formation rate was recorded at 96 h.

### SRS Microscopy System Setup

The SRS microscope is illustrated in Figure S10. The fundamental 1031 nm beam (∼2 ps) was used as the Stokes, and the wavelength tunable output (700–990 nm) was used as the pump. The pump beam was tuned to 792.7 and 785.9 nm for the two Raman bands at 2900 and 3010 cm^–1^. The Stokes beam was intensity modulated by an electro-optical modulator (EOM) at 80 MHz and collinearly combined with the pump beam through a dichroic mirror (650 nm cutoff, Thorlabs). The combined beam was delivered to the laser scanning microscope (BX51WI, Olympus) and focused onto the samples with an objective (UPLSAPO 60XWIR, NA 1.2 water, Olympus). The pump beam was detected by a homemade photodiode (PD), and then the SRS signal was extracted by a lock-in amplifier (LIA). For cell imaging, each field of view (FOV) was imaged with a size of 400 × 400 pixel^2^ (120 × 120 μm^2^) with lateral resolution of ∼300 nm, and a 10 μs pixel dwell time. Each FOV was averaged three times. 3D SRS images of two channels (2900 cm^–1^, 3010 cm^–1^) were acquired as *z*-stacks over 60 μm depth with a step size of 1 μm. For standard oil film imaging (Figure S4), partial air contrast in FOV was imaged with a size of 400 × 400 pixel^2^ (120 × 120 μm^2^) with lateral resolution of ∼300 nm, and 3D SRS images of two channels (2900 cm^–1^, 3010 cm^–1^) were acquired as *z*-stacks over 72 μm depth with a step size of 1 μm. The result of the standard sample (Figure S4F) indicates that the degree of unsaturation calculation result will not be affected by the imaging depth.

Hyperspectral imaging is performed by tuning the pump beam. When the Stokes beam was tuned to 1031 nm, the pump beam was tuned from 799.1 to 783.4 nm to cover the entire 2800–3050 cm^–1^ vibration region. In practice, a stack of 50 images was obtained at different pump-Stokes temporal delays corresponding to 2800–3050 cm^–1^ in 5 cm^–1^ steps over a 120 μm × 120 μm area, with 0.3 μm pixel size and a pixel dwell time of 10 μs.

### Spontaneous Raman Spectroscopy

A laser at 532 nm was used as the excitation beam for Raman spectral acquisition. Confocal Raman spectral analysis from individual points was performed. Acquisition time for a typical spectrum from standard fatty acid sample was 60 s, with the beam power maintained around 15 mW at the standard fatty acid sample. The spectra were analyzed using the software Origin 2019b. The background was removed manually, and the peak height was measured.

### Image Processing

Images were processed by Matlab. The work flow of image processing is illustrated in Figure S3. The original 3D tiff image stacks are loaded as 3D matrices. First, the background signal (baseline) of the same layer is subtracted from the signal of each layer in the image 3D stack to obtain the real signal. Gaussian filtering is performed to smooth object boundaries and reduce jagged edges. In order to eliminate the impact of signal attenuation and uneven illumination, we used an adaptive threshold method to extract LDs in each 2D layer and obtain the representative lipid binary image of the spatial distribution of LDs. For the 3D stack of the lipid channel, each LD is marked to obtain their distribution attributes in 3D space by connectivity analysis. The volume, number, and aggregation of all LDs in the entire embryo then are measured. At the same time, the ratio value of the SRS signals at 3010 and 2900 cm^–1^ (3010/2900 cm^–1^) is used to reflect the level of lipid unsaturation, and the ratio is proportional to the level of unsaturation. By changing the threshold of 2900 cm^–1^ channels, we can obtain the overall morphology of the cell. Morphological opening operation and hole filling then were implemented to eliminate holes caused by the heterogeneous brightness distribution in some cells in the binary image. Three-dimensional visualization of the merged images of lipid unsaturation distribution and cell morphology was achieved using Fiji ImageJ.

For the segmentation of the two cells during the 2C stage, to overcome the inaccuracies of the watershed algorithm, we employed an image segmentation approach based on concavity point detection and cluster analysis, leveraging the characteristics of the connecting region between the two cells. After binarizing the cell morphology, we extracted the image contours and performed a concave point detection algorithm to obtain the distribution of concave points. By using the *k*-means clustering algorithm, the position centers of the two largest concave point aggregates can be obtained. The line connecting these two centers was then employed for cell segmentation.

### Statistical Analysis

For statistical analysis, data were first tested for normality. If the data are non-Gaussian, a nonparametric Mann–Whitney U-test was performed. Otherwise, a two-tailed student’s *t* test was performed. Significant differences were considered at *p < 0.05, **p < 0.01, n.s. means not significant, data shown as mean ± sem. To clearly plot the relationship between lipid unsaturation level and aggregate size, we divided all lipid aggregates into intervals based on their size, with intervals of 5 μm^3^, and then calculated the average unsaturation of all LD aggregates in each interval as scatter points. Scatter plots show the correlations between the mean lipid unsaturation level and aggregate size at different stages of development. The line and shading in the graph represent the linear regression and 95% confidence interval, respectively.

## Data Availability

All data generated or analyzed during this study are available upon request.

## Supporting Information Available

Supplemental figures; embryo lipid unsaturation level plotted by 3D SRS imaging (Figure S1); spontaneous Raman and SRS spectra of standard fatty acid (Figure S2); 3D image processing (Figure S3); 3D imaging of standard oil film and calculation of unsaturation at different imaging depths (Figure S4); lipid composition difference between WT and Scd1_KD (Figure S5); single-cell segmentation process (Figure S6); lipid size and unsaturation distribution in oocytes and early embryonic stages (Figure S7); lipid unsaturation distribution and SRS spectrum of a single plane in oocytes, and early embryonic stages (Figure S8); lipid composition difference between normal and progeria (LMNA^–/–^) oocytes (Figure S9); and schematic representation of SRS microscopy experimental setup (Figure S10) (PDF)
