# Supplementary material for: Lipid Metabolic Heterogeneity during Early Embryogenesis Revealed by Hyper-3D Stimulated Raman Imaging
Source: Chem Biomed Imaging. 2024 Oct 4;3(1):15–24. doi: 10.1021/cbmi.4c00055 (PMC11775849; doi:10.1021/cbmi.4c00055)
Supplement: Supplementary file 1 — im4c00055_si_001.pdf [file im4c00055_si_001.pdf]

Support information for
Lipid metabolic heterogeneity during early embryogenesis revealed by hyper-3D stimulated Raman imaging

Jie Huang[1,2+], Ling Zhang[3,4+], Ninghui Shao[1], Yongqing Zhang[6], Yuyan Xu[3,4], Yihui Zhou[1], Delong Zhang[6,7], Jin Zhang[3,4,5*], Hyeon Jeong Lee[1,7*]

[1]College of Biomedical Engineering & Instrument Science; Key Laboratory for Biomedical Engineering of Ministry of Education, Zhejiang University, Hangzhou, China 310058

[2]Zhejiang Polytechnic Institute, Polytechnic Institute, Zhejiang University, Hangzhou, China 310058

[3]Liangzhu Laboratory, Zhejiang University, Hangzhou, China 311121

[4]Center for Stem Cell and Regenerative Medicine, Department of Basic Medical Sciences, and Bone Marrow Transplantation Center of the First Affiliated Hospital, Zhejiang University School of Medicine, Hangzhou, China 310058

[5]Center of Gene and Cell Therapy and Genome Medicine of Zhejiang Province, Hangzhou, China 310058

[6]Interdisciplinary Centre for Quantum Information; Zhejiang Province Key Laboratory of Quantum Technology and Device; Department of Physics, Zhejiang University, Hangzhou, China 310027

[7]MOE Frontier Science Center for Brain Science & Brain-Machine Integration, Zhejiang University, Hangzhou, China 310027

[+]These authors contributed equally

[*]Corresponding authors. Email: zhgene@zju.edu.cn, hjlee@zju.edu.cn

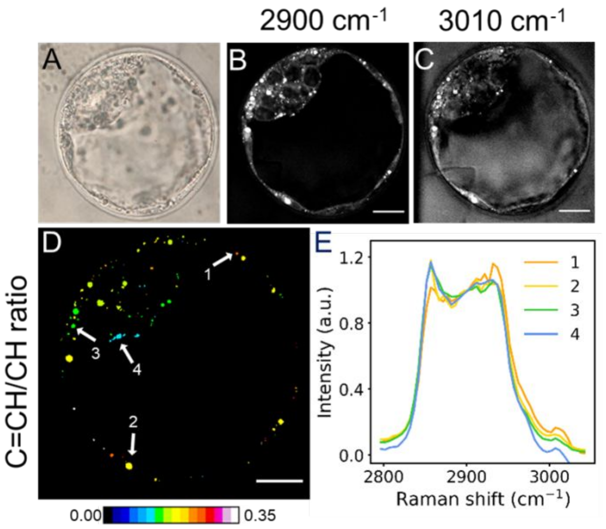

Figure S1. **Embryo lipid unsaturation level plotted by 3D SRS imaging. (A)** Bright-field image of blastocyst embryo. **(B)** Raw SRS image at 2900 cm^{-1}. **(C)** Raw SRS image at 3010 cm^{-1}. **(D)** Lipid signal ratio image between 3010 and 2850 cm^{-1}. **(E)** SRS spectral of the position indicated by arrows in **(D)**. Scale bar: 20 µm.

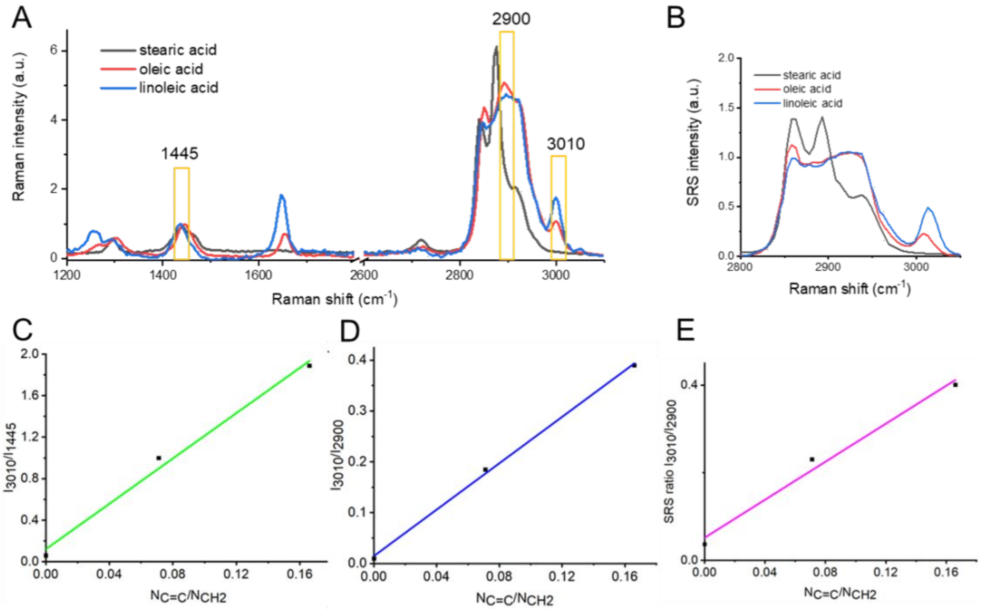

Figure S2. **Spontaneous Raman and SRS spectra of standard fatty acid. (A)** Spontaneous Raman, **(B)** SRS spectra of stearic acid, oleic acid and linoleic acid. **(C)** Dependence of the ratio of Raman spectral peaks at 3010 cm^{-1} and 1445 cm^{-1} on the molecule mass unsaturation $N_{C=C}/N_{CH2}$, straight line is a fit of this data. **(D)** Dependence of the ratio of Raman spectral peaks at 3010 cm^{-1} and 2900 cm^{-1} on the molecule mass unsaturation $N_{C=C}/N_{CH2}$, straight line is a fit of this data. **(E)** Dependence of the observed ratio of SRS spectral peaks at 3010 cm^{-1} and 2900 cm^{-1} on the molecule mass unsaturation $N_{C=C}/N_{CH2}$, straight line is a fit of this data.

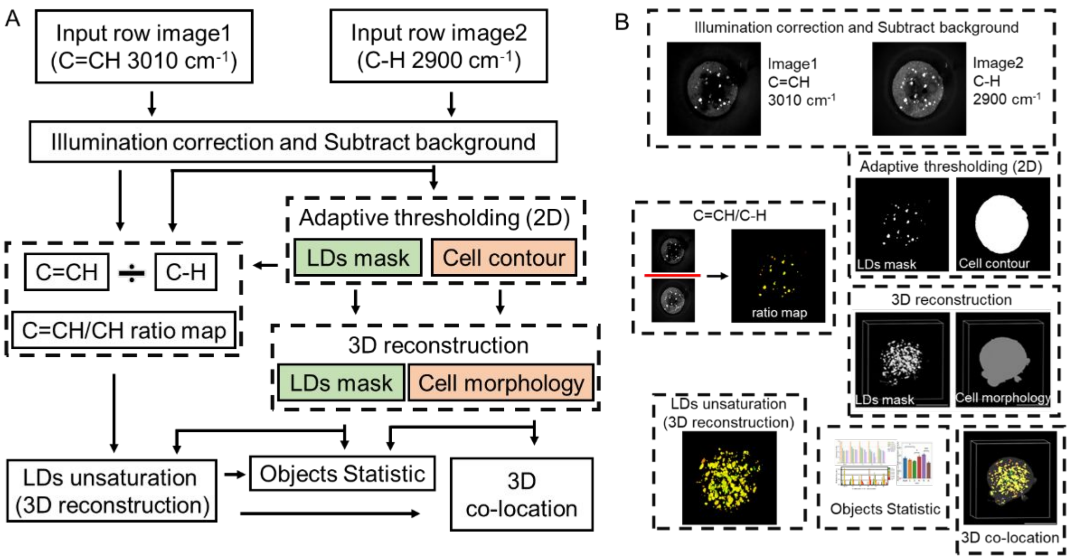

Figure S3. **3D image processing process. (A)** Image processing flowchart. **(B)** Examples of image processing as outlined in **(A)**.

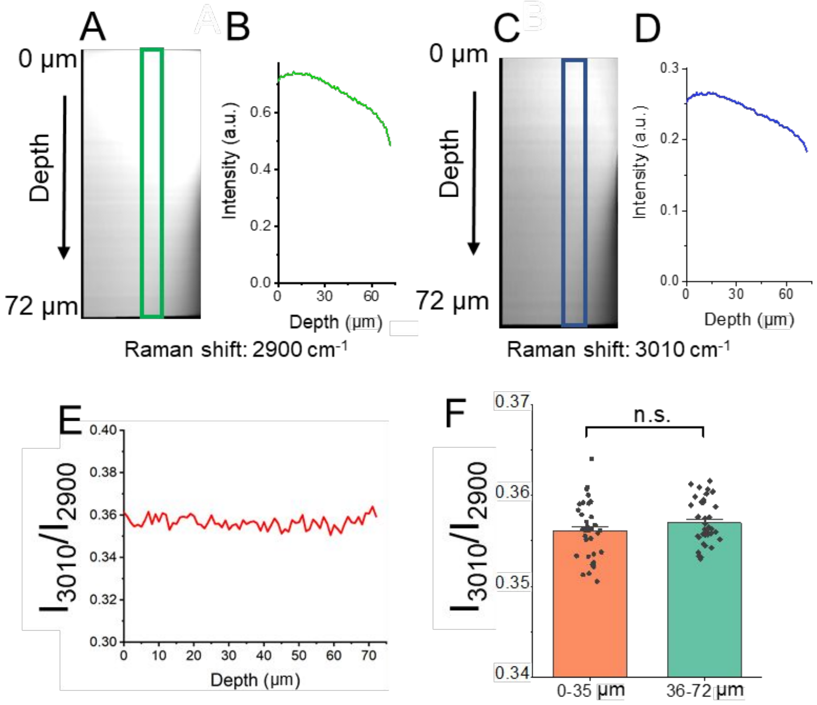

Figure S4. **3D imaging of standard oil film and calculation of unsaturation at different imaging depths. (A)** 3D image of Raman shift in 2900 cm^{-1}. **(B)** Curve of SRS intensity in 2900 cm^{-1} with depth. **(C)** 3D image of Raman shift in 3010 cm^{-1}. **(D)** Curve of SRS intensity in 3010 cm^{-1} with depth. **(E)** Variation of the ratio of SRS intensity in 2900 cm^{-1} and 3010 cm^{-1} with depth. **(F)** Variance analysis of ratio results with depth between 0-35 μm and 36-72 μm.

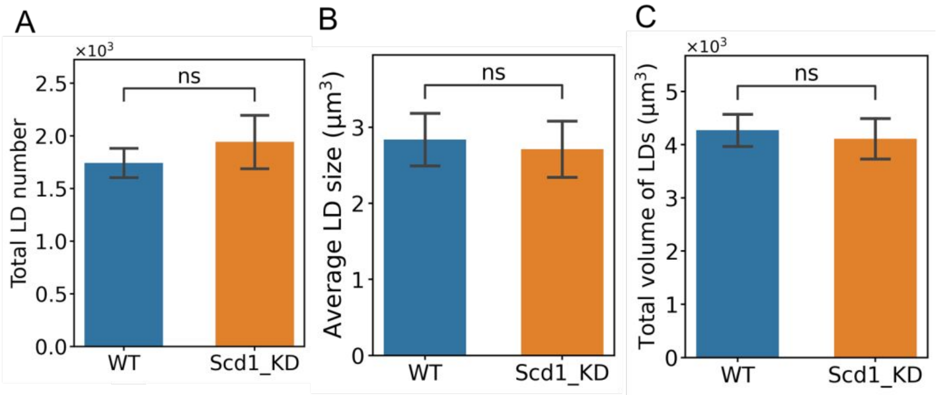

Figure S5. **Lipid composition difference between WT and Scd1_KD. (A)** Number of LDs between two groups. **(B)** LD size distribution between two groups. **(C)** Total volume of LDs between two groups. BC: n=11, Scd1_KD: n=10, Mann–Whitney U-test, n.s. non-significant, *P < 0.05, **P < 0.01, data shown as mean ± s.e.m. Scale bar, 50 μm.

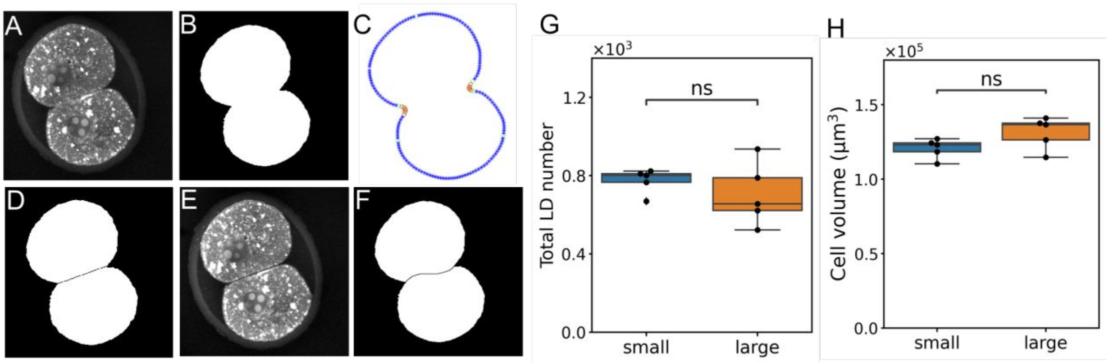

Figure S6. **Single-cell segmentation process. (A)** Raw SRS image at 2900 cm^{-1}. **(B)** Binary image of cell contour in **(A)**. **(C)** Obtaining segmentation points through concave point detection and clustering analysis. **(D)** Segmentation result of binary mask. **(E)** Segmentation result of raw image. **(F)** Watershed algorithm segmentation results. **(G)** Number of LDs between two groups. **(H)** Cell volume of single cell of 2C stage.

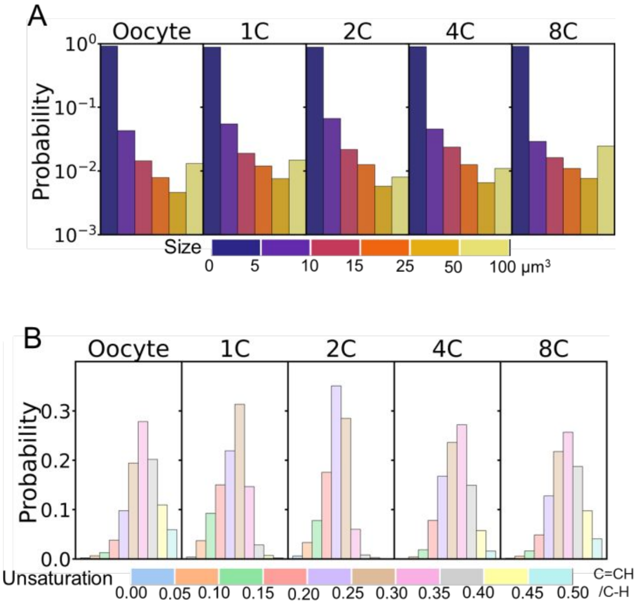

Figure S7. **Lipid size and unsaturation distribution in oocytes and early embryonic stages. (A)** LD size distribution at different embryonic stages. **(B)** Frequency distribution of LDs unsaturation level. Oocyte: n=6, 1C: n=11, 2C: n=5, 4C: n=8, 8C: n=7.

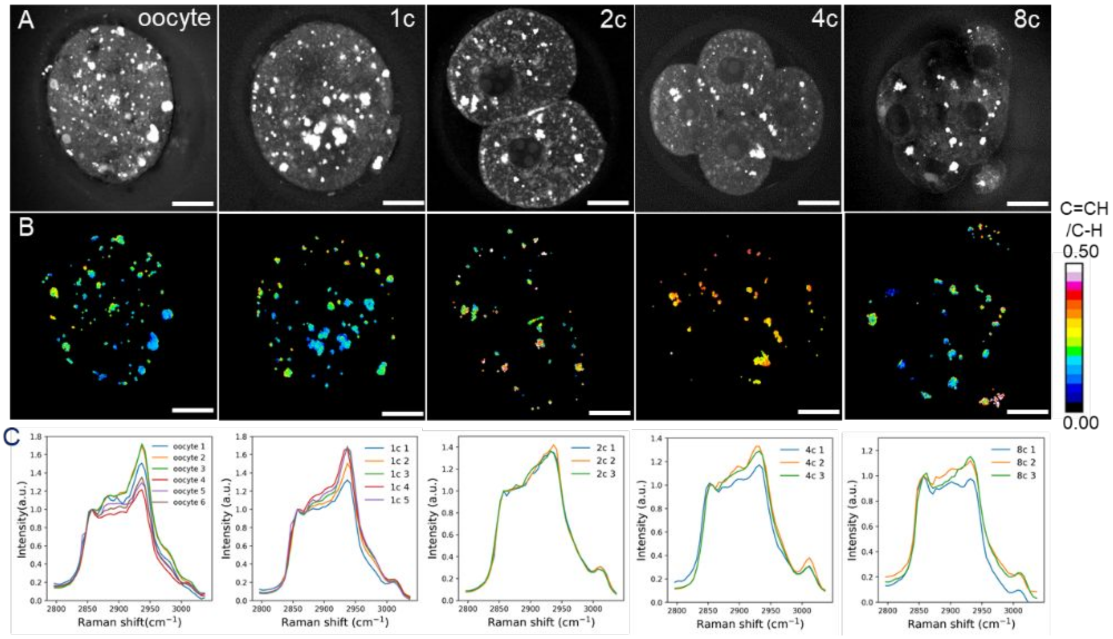

Figure S8. **Lipid unsaturation distribution and SRS spectrum of a single plane in oocytes, and early embryonic stages. (A)** Representative raw SRS image at 2850 cm^{-1} (lipid) of oocytes, and early embryonic stages. **(B)** Corresponding intensity ratio image between 3010 and 2850 cm^{-1} in **(A)**. **(C)** SRS spectra from LDs in oocytes, and early embryonic stages. Each spectrum represents the average spectrum of all LDs in a single cell. Oocyte: n=6, 1C: n=5, 2C: n=3, 4C: n=3, 8C: n=3. Scale bar: 20 μm.

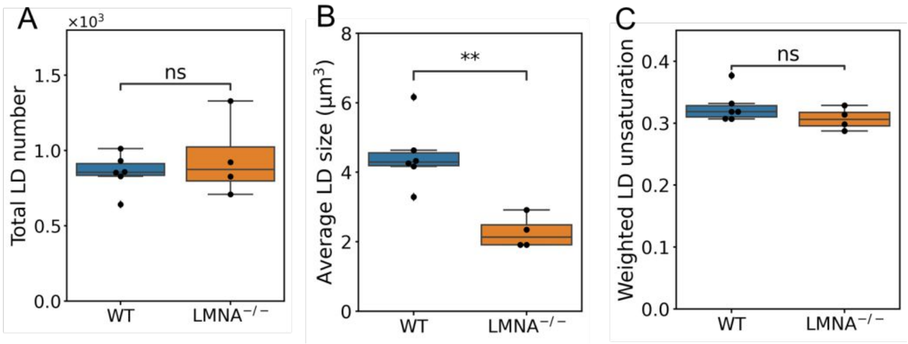

Figure S9. **Lipid composition difference between normal and progeria (LMNA$^{-/-}$) oocytes. (A)** Number of LDs between two groups. **(B)** LD size distribution between two groups. **(C)** Unsaturation of embryo between two groups of embryos. WT: n=6, LMNA$^{-/-}$: n=4, Each point represents one cell, Mann–Whitney U-test, n.s. non-significant, *P < 0.05, **P < 0.01, data shown as mean ± s.e.m.

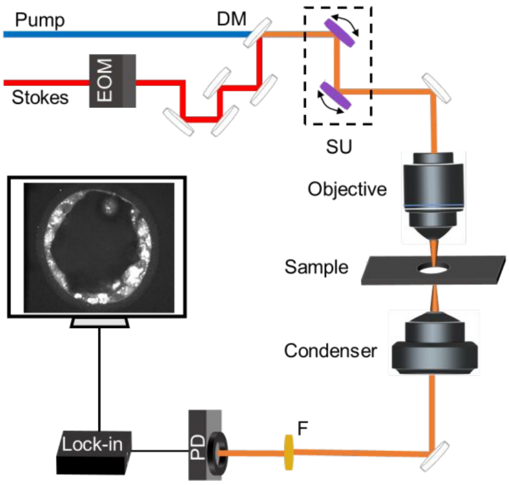

Figure S10. **Schematic representation of SRS microscopy experimental setup.** EOM: electro-optic modulator; DM: dichroic mirror; SU: scanning unit; F: filter; PD: photodiode.
